# A Rare Case of Hepatocellular Carcinoma Presenting as a Massive Abdominal Hematoma and Shock: A Case Report

**DOI:** 10.7759/cureus.34588

**Published:** 2023-02-03

**Authors:** Kaveh Mozafari, Dejeau P Pyfrom, Joancy M Archeval-Lao, Shanynn Santos, Frederick Tiesenga

**Affiliations:** 1 Surgery, West Suburban Medical Center, Oak Park, USA; 2 Medicine, St. George's University School of Medicine, St. George, GRD; 3 Medicine, Saint James School of Medicine, Park Ridge, USA; 4 Surgery, West Suburban Medical Center, Chicago, USA; 5 Medical Education, St. George's University School of Medicine, True Blue, GRD; 6 General Surgery, West Suburban Medical Center, Chicago, USA

**Keywords:** abdominal, hematoma, hepatocellular carcinoma, abdominal gunshot, surgery general, hypovolemic shock, abdominal hematoma, hepatocellular carcinoma (hcc)

## Abstract

Hepatocellular carcinoma (HCC) has an affluent blood supply stemming from the hepatic artery. Subsequent spontaneous tumor rupture can lead to massive abdominal hematoma and shock, a rare fatal gastrointestinal incident. The diagnosis of rupture is complicated, with most patients presenting with abdominal pain and shock. Prompt correction of hypovolemic shock is the primary goal of treatment. This rare case presents a 75-year-old male who presented to the emergency department because of abrupt and increasing abdominal pain after a meal. Laboratory data revealed elevated alanine aminotransferase, aspartate aminotransferase, and alpha-fetoprotein levels. Immediate computed tomography demonstrated a defect in the right ventral abdominal wall. The patient underwent an emergency exploratory laparotomy. Despite massive intra-abdominal adhesions, the identified source of bleeding was from the left lobe of the liver at the base of the lesser sac above the pancreas. There was a maximum effort to cease bleeding and minimize blood loss. An ensuing biopsy of the liver revealed HCC. After improving, the patient received instructions to follow up on an outpatient basis. Two months after surgery, the patient endorses no complications. The success outlined in this case highlights the essence of prompt action in an emergency, which delineates the significance of surgical experience in handling unorthodox patient presentations.

## Introduction

Abdominal hematomas are uncommon and often mimic other acute abdominal disorders [1]. Hematomas occur because of an accumulation of blood secondary to an injury to the epigastric vessels, their perforating branches, or a solid organ [2]. Hematomas have been commonly associated with elderly females with a history of anticoagulant use or those who suffered from trauma [1,3]. Despite the established association, some hematomas can occur spontaneously, with no predispositions [4]. For example, a spontaneous hemorrhage can occur because of visceral injuries in the liver, spleen, kidneys, or adrenal glands [5].

Hepatocellular carcinoma (HCC) is an uncommon but fatal cause of abdominal hemorrhage, with an incidence of up to 15% in patients diagnosed with the disease [6]. It is associated with a poor prognosis, especially in the setting of cirrhosis and severe coagulopathy [6]. It requires prompt treatment to prevent dire consequences, like uncontrolled hemorrhage and death [7]. While a hematoma can resolve with no intervention, in rare cases, patients need surgery when the hematoma is large or progressing.

Because of the infrequency and imitative nature, it is challenging to diagnose abdominal hematomas, and clinicians often mistake them for inflammatory diseases [1,3]. Therefore, there is a need for accurate diagnosis. Computed tomography (CT) and other imaging modalities can support the diagnosis by identifying the hallmark features of active bleeding. Naturally, clinicians should emphasize identifying the source promptly and determining the etiology of the bleeding to prevent drastic complications. This report presents an elderly male with a sudden onset of abdominal pain and hypotension who underwent emergent surgical treatment after surgeons discovered an abdominal hematoma secondary to a spontaneous hemorrhage of HCC on CT.

## Case presentation

Here, we describe a case of a 75-year-old male who presented to the emergency department because of abrupt and increasing abdominal pain after having a meal. The patient had a past medical history of hypertension and hepatitis. The patient became hemodynamically unstable en route to the hospital, with his systolic blood pressure dropping below 50 mmHg. When he arrived in the emergency room, he was experiencing non-bloody, nonbilious emesis. His blood pressure was 69/47 mmHg, and his heart rate was 84 beats per minute. The patient received immediate fluid resuscitation. There was moderate abdominal distension and diffuse tenderness. Laboratory data revealed an elevation of alanine aminotransferase (ALT) at 232 IU/L, aspartate aminotransferase (AST) at 171 IU/L, and alpha-fetoprotein (AFP) at 3000 ng/mL levels. In addition, the patient tested positive for hepatitis C antigen but negative for hepatitis C RNA.

The patient got an immediate CT of the abdomen. Imaging revealed a defect in the right ventral abdominal wall with multiple metallic fragments consistent with shrapnel that extended into the right iliopsoas muscle. The liver had multiple hypodense masses with a metallic fragment lodged in the caudate lobe. This corresponds with his past medical history. Approximately 50 years prior, the patient sustained a gunshot wound to the abdomen and underwent extensive surgery for an abdominal wall and recurrent small bowel obstruction repair.

**Figure 1:**
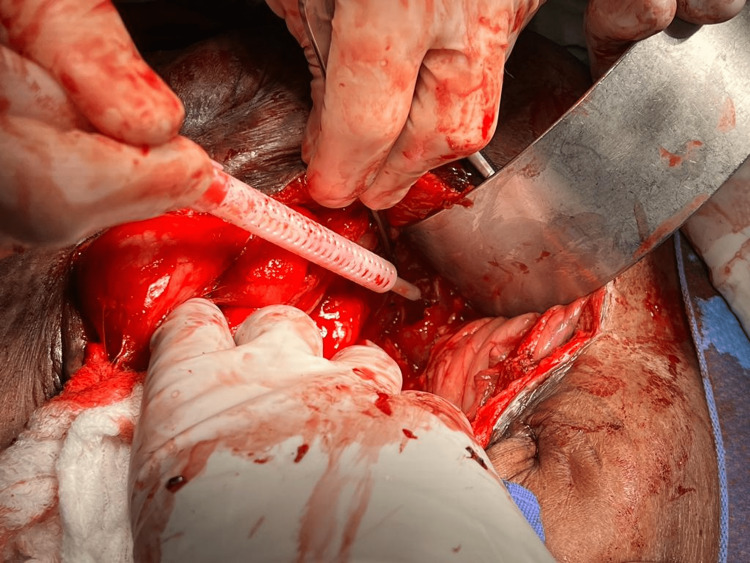
Emergency exploratory laparotomy with massive hematoma.

There was also an intraperitoneal hematoma without evidence of a ruptured aorta. Upon confirmation of diagnosis, the patient underwent an emergency exploratory laparotomy (Figure 1).

The surgeons opened the abdomen and found the lesser sac to have massive blood in the space. They evacuated the blood from the lesser sac and found that a portion of the liver was bleeding. During this time, the patient became hemodynamically unstable. Maximum effort to cease bleeding began; manual pressure, sterile compressed sponge, and powder hemostat proved helpful. Eventually, surgeons controlled the bleeding after placing liver sutures. He received six units of packed red blood cells, with subsequent improvement. Next, the surgeons biopsied the part of the liver that had been bleeding and sent it for pathological examination. The biopsy revealed atypical hepatocytes and dilated blood vessels, favoring a diagnosis of HCC. The patient’s postoperative course was without further complications, with signs of significant improvement. His ALT, AST, and AFP levels had improved significantly. He continues to follow up in an outpatient setting and endorses feeling well with no nausea or pain.

## Discussion

Several factors can lead to abdominal hematomas; however, spontaneous HCC rupture is a very plausible cause of hemorrhage in this patient. The exact pathophysiology is unknown, but some researchers have proposed several mechanisms to explain the rupture mechanism [8]. The first hypothesis suggests that a rupture can occur secondary to vascular injuries. An increased collagenase expression in injured vessels degrades type IV collagen, which is essential for mechanical stability [9]. These arteries become stiff and can rupture. Second, research suggests that venous congestion caused by a tumor or chronic liver disease could lead to an HCC rupture. When the hepatic vein becomes occluded, it can prevent blood from flowing out of the liver. This causes more blood to flow into the tumor, increasing intra-tumoral pressure and making the tumor more prone to rupture [8]. Other hypotheses base the tumor’s susceptibility to rupture on the hepatic parenchyma surrounding the HCC, size, and location. Normal parenchyma surrounding the HCC can help prevent the rupture of the tumor, but thinner parenchyma can make the tumor more prone to rupture. The tumor's location also plays an important role; if the HCC is in a subscapular location, it can rupture earlier than an HCC tumor in the center of the liver. If the HCC is in the left lobe of the liver, it has a higher risk of rupture because the left lobe has a smaller area for space-occupying lesions [8].

Spontaneous hepatic hemorrhage secondary to rupture of HCC is challenging to diagnose and treat. Not detecting and controlling early can lead to shock and death [10]. As it has a poor prognosis, it must be a differential diagnosis in patients with higher levels of liver dysfunction [11]. Despite the poor prognosis, some studies show that emergency laparotomy is detrimental to patient survival [12]. One study by Chedid et al. [12] outlined three separate cases of spontaneous hemorrhages because of HCC rupture. All patients had a similar background and clinical manifestation, including a history of chronic liver disease, specifically cirrhotic liver, as in our patient. They admitted patients who presented with abdominal pain and hypotension for an emergent laparotomy with varying degrees of success.

The patient’s liver had become enlarged and cirrhotic in the first case discussed. Successive pathological workup revealed HCC and cirrhosis. Similarly, the second patient’s imaging revealed a tumor on the right side of the liver with free peritoneal fluid. This patient underwent an elective right trisegmentectomy, and subsequent histology confirmed HCC and cirrhosis. However, 16 months later, the patient returned with diffuse abdominal pain with signs of hypovolemia, chronic liver disease, and a tense abdomen. CT revealed an encapsulated hematoma, and laparotomy revealed a hematoma in the remaining left liver. Biopsy showed that the HCC had spread to the left lobe. The last case showcased a patient with a history of viral hepatitis C and cirrhosis. Imaging revealed large intra-abdominal fluid and an ulcerated lesion in the right hepatic lobe. Sadly, the patient died from hypovolemic shock with external bleeding through the abdominal drain, which she developed during the immediate postoperative period of the right hepatectomy.

The patient's acute presentation suggests an HCC rupture caused his hematoma. Per past literature, our patient’s symptoms mirror those of other patients who presented for HCC rupture. Seemingly, our patient presented with abnormal liver function tests, severe hypotension, and abdominal distension. Diagnosing ruptured HCC in patients is challenging because there are various presentations. However, most studies describe shock as the most critical indicator of rupture [13]. Optimal treatment is required as rupture of HCC is a life-threatening complication. Even though there is a plethora of treatment methods, an emergent laparotomy in the present case proved essential in the treatment of rupture and diagnosis of HCC.

## Conclusions

In brief, abdominal hematomas often have varying presentations yet can rarely be associated with cancer. HCC has a rich blood flow and can lend itself to a more aggressive course if ruptured. Although trauma and chronic diseases can also cause hematomas, the overall goal is to diagnose and treat presenting patients promptly. Although one would assume that an exploratory laparotomy is the most successful course of action with hematoma, treatment is patient-dependent. With this patient, an exploratory laparotomy was critical to the patient’s survival, but it must be determined what will provide the best outcome for the patient. The early diagnosis of hematomas will improve outcomes and allow the management of injury before it reaches stages that require emergent operations.
